# Foliar brassinosteroid analogue (DI-31) sprays increase drought tolerance by improving plant growth and photosynthetic efficiency in lulo plants^☆^

**DOI:** 10.1016/j.heliyon.2022.e08977

**Published:** 2022-02-19

**Authors:** Cristian Camilo Castañeda-Murillo, Javier Gustavo Rojas-Ortiz, Alefsi David Sánchez-Reinoso, Cristhian Camilo Chávez-Arias, Hermann Restrepo-Díaz

**Affiliations:** Universidad Nacional de Colombia, Sede Bogotá, Facultad de Ciencias Agrarias, Departamento de Agronomía, Carrera 30 No. 45-03, Bogotá, 111321, Colombia

**Keywords:** Drought stress, Andean fruit species, Leaf photosynthesis, Foliar spray, Malondialdehyde, Plant hormones

## Abstract

The use of agronomic alternatives such as plant hormone sprays has been considered a tool to mitigate drought stress. This research aimed to evaluate the use of foliar brassinosteroid analogue DI-31 (BRs) sprays on plant growth, leaf exchange and chlorophyll *a* fluorescence parameters, and biochemical variables in lulo (*Solanum quitoense* L. cv. *septentrionale*) seedlings grown under drought stress conditions. Seedlings were grown in plastic pots (3 L) using a mix between peat and sand (1:1 v/v) as substrate. Lulo plants were subjected to drought stress by suppressing 100% of the water needs at 30–37 and 73–80 days after transplanting (DAT). Foliar BRs analogue (DI-31) sprays were carried out at four different rates (0, 1, 2, 4, or 8 mL of analogue per liter) at different times (30, 33, 44, 60, 73, and 76 DAT). Drought stress caused a reduction in the F_v_/F_m_ ratio, leaf gas exchange properties, total biomass, and relative water content. Foliar DI-31 sprays enhanced leaf photosynthesis in well-watered (WW) (∼10.7 μmol m^−2^ s^−1^) or water-stressed plants (WS) (∼6.1 μmol m^−2^ s^−1^) when lulo plants were treated at a dose of 4 and 8 mL·L^−1^ compared to their respective controls (0 mL·L^−1^ for WW: 8.83 μmol m^−2^ s^−1^ and WS: 2.01 μmol m^−2^ s^−1^). Also, DI-31 sprays enhanced the photochemical efficiency of PSII, and plant growth. They also increased the concentration of photosynthetic pigments (TChl and Cx + c) and reduced lipid peroxidation of membranes (MDA) under drought conditions. The results allow us to suggest that the use of DI-31 at a dose of 4 or 8 mL·L^−1^ can be a tool for managing water stress conditions caused by low water availability in the soil in lulo-producing areas to face situations of moderate water deficit at different times of the year.

## 1 Introduction

Lulo or naranjilla (*Solanum quitoense* Lam.) is an important tropical fruit tree belonging to the Solanaceae family and its origin is in Ecuador and Colombia (Ávila et al., 2019). Its fruits are characterized by their refreshing and intense aroma, with potential interest for international markets, such as the United States and Europe (Forero et al., 2016; Ávila et al., 2019). Lulo is grown at altitudes between 1000 and 1990 m above sea level in Colombia, Ecuador, Panama and Costa Rica (Fló rez – Velasco et al., 2015; Ramírez et al., 2018). In Colombia, lulo crops had a cultivated area of 8,821 ha with an average yield of 10.1 t·ha^−1^ in 2018 (Agronet, 2021).

Climate change poses increasingly severe risks to crop production as it affects weather patterns, resulting in extreme heat events, frequent frosts, waterlogging, and drought (Dahal et al., 2019; Menezes– Silva et al., 2019). Drought stress is one of the principal limitations to agricultural productivity worldwide (Nawaz et al., 2015). World regions most affected by drought include South and Central Asia, South America, Central Europe, and the Southeastern United States (Goñi et al., 2018). Drought events can also be associated with climate variability phenomena such as El Niño-Southern Oscillation (ENSO), generating more frequent and intense drought periods (Zambrano Mera et al., 2018; Oñ ate – Valdivieso et al., 2020). In Colombia, ENSO phenomena cause reductions in rainfall and increases in average temperatures (Sá nchez – Reinoso et al., 2018). As reported by the Organization for Economic Cooperation and Development (OECD) (2014), decreases in the average annual precipitation rate of 4 mm year^−1^ are projected, which may generate extreme drought events in Colombia for the year 2100. A transition can be expected from a semi-humid to a semi-arid climate in the main lulo-producing areas in Colombia (Andean region) (OECD, 2014; Agronet, 2021).

Drought can influence plant metabolism, growth, and productivity (Abid et al., 2018; Kumar et al., 2018; Liang et al., 2020). Net photosynthesis (P_*N*_) is the physiological process most affected by this type of stress (Li et al., 2018; Talaat, 2020). Photosynthetic deterioration during water scarcity is mainly caused by stomatal (stomatal closure due to CO_2_ reduction) or non-stomatal (decrease in chlorophyll content, inhibition of Rubisco, and photochemical efficiency of PSII) limitations that alter carbohydrate metabolism and dry matter partitioning (Zahoor et al., 2017; Li et al., 2018; Zhao et al., 2020). Liu et al. (2015) observed a decrease in growth, P_*N*_, stomatal conductance (g_s_), and chlorophyll content of tomato (*Solanum lycopersicum* L.) plants under conditions of drought stress. The above has allowed concluding that the estimation of gas exchange parameters such as P_*N*_, g_s_, and transpiration (*E*) is a useful technique to evaluate plant responses to drought and its mitigation strategies (Hu et al., 2013; Gill et al., 2017).

Leaf photosynthetic pigments such as carotenoids (Cx + c) and total chlorophyll (TChl) have become important elements in monitoring drought stress and quantifying agronomic management techniques in plants (Chen et al., 2016; Shah et al., 2017). Batra et al. (2014) recorded a gradual decrease in the TChl content and an increase in Cx + c values of three genotypes of mung bean (*Vigna radiata* L.) exposed to drought. However, the application of agronomic management strategies such as mineral fertilizers, compost, and plant growth-promoting rhizobacteria (PGPR) showed an increase in TChl of 82% in wheat plants under conditions of drought stress (Kanwal et al., 2017).

Chlorophyll fluorescence parameters are also a rapid, complementary, and non-destructive technique to estimate the effects of stress (including drought) on the functioning of the photosynthetic system (Liu et al., 2019). Chlorophyll fluorescence parameters such as quantum efficiency of PSII (F_v_/F_m_), photochemical quenching (qP), and non-photochemical quenching (NPQ) have been used as indicators of tolerance and acclimatization to drought stress conditions in cotton (*Gossypium hirsutum* L.) plants (Singh et al., 2014) and Moso Bamboo (*Phyllostachys edulis*) (Wu et al., 2018). Raja et al. (2020) recorded drops in F_v_/F_m_ and qP values, and higher NPQ in tomato plants under a 10-day drought period. In contrast, Pourghasemian et al. (2020) recorded an increase in F_v_/F_m_ after the use of biostimulants (salicylic acid and beeswax waste and licorice extracts) as effective agronomic practices in sesame (*Sesamum indicum* L.) plants under water deficit conditions.

Drought stress also causes the buildup of reactive oxygen species (ROS). High levels of these molecules are harmful since they damage membranes, proteins, chlorophyll, and nucleic acids in plants (Yuan et al., 2015; Talaat, 2020). Malondialdehyde (MDA) is generated from the lipid peroxidation of plant cell membranes and has been used as a biochemical indicator of membrane damage under drought conditions (Cheng et al., 2018; Liang et al., 2019). MDA has also been used as a biochemical marker to evaluate the effect of agronomic strategies for optimizing crop resilience to water deficit conditions (Gill et al., 2017; Gou et al., 2017). These same authors recorded a lower MDA content after the use of agronomic strategies such as the application of nitrogen fertilizers or plant hormones, mitigating stress caused by water shortage in barley (*Hordeum vulgare* L.) and maize (*Zea mays* L.) plants, respectively.

Exogenous applications of plant hormones or biostimulants are also an agronomic strategy used as an adaptation method to improve crop productivity and water use efficiency under drought conditions (Hussain et al., 2018; Pourghasemian et al., 2020). Foliar applications of brassinosteroids (BRs) have been considered an alternative to lessen the damage caused by drought conditions in plants (Lima and Lobato, 2017; Khamsuk et al., 2018). Exogenous BRs mitigate the negative effects of this type of stress on growth, gas exchange parameters (increasing P_*N*_, g_s_, and water use efficiency (WUE)), photosynthetic pigment contents, or chlorophyll fluorescence measurements such as F_v_/F_m_ in cultivated plants (Khamsuk et al., 2018; Lima and Lobato, 2017; Talaat, 2020). Foliar applications of BRs help plants to cope with drought stress by improving water relations and plant growth, leaf gas exchange parameters (P_*N*_, g_s,_ and *E*), photosynthetic pigment contents (TChl and Cx + x), and chlorophyll fluorescence parameters such as F_v_/F_m_, qP and NPQ in chili pepper [*Capsicum annuum* L. var. *frutescens* (L.) Kuntze] (Khamsuk et al., 2018) and cowpea [*Vigna unguiculata* (L.) Walp.] plants (Lima and Lobato, 2017). Yuan et al. (2010) also found that this plant hormone can reduce oxidative damage reflected in a lower MDA accumulation in tomato plants. One of the most used BRs is 24-Epibrassinolide (EBL) since it improves the growth, yield, and photosynthetic parameters of plants under drought conditions (Tanveer et al., 2019; Khan et al., 2021). However, the application of this plant hormone results in increased costs to producers (Serna et al., 2015). Therefore, the use of brassinosteroid analogues (DI-31) results in a cost-efficient tool to alleviate the negative effects of abiotic stress (Pérez-Borroto et al., 2021). BRs analogues exhibit greater biological activity and longer average life under field conditions (Sasse, 2003). These characteristics, along with a growth-promoting effect in different plant species and lower cost, make these compounds a good option to replace some of the most commonly used hormones such as EBL (Pérez-Borroto et al., 2021). DI-31 has been reported to improve crop yield and increase the photosynthetic rate of plant species grown under field conditions (Serna et al., 2012a, 2012b, 2015).

Different studies on plant ecophysiology have been mainly focused on the effect of the water stress (drought or waterlogging) or light intensity on leaf gas exchange and chlorophyll fluorescence parameters, morphoanatomical changes, and plant growth (Medina et al., 2006; Cardona et al., 2016; Sá nchez-Reinoso et al., 2019). The literature reports the use of foliar nitrogen sprays to enhance lulo tolerance to water stress (waterlogging) (Fló rez – Velasco et al., 2015). Research on the use of agronomic management strategies to mitigate drought stress conditions is scarce in lulo. Additionally, no studies have reported the use of a brassinosteroid analogue on the growth and physiological behavior of an Andean fruit species such as lulo under drought stress. This study hypothesized that the exogenous application of BRs analogue DI-31 would be an agronomical tool to alleviate the negative impacts of drought by enhancing physiological and biochemical traits in lulo plants. Therefore, the aim of this research was to determine if DI-31 can lessen the negative effects caused by water deficit on plant growth, leaf gas exchange parameters, photosynthetic pigments, chlorophyll fluorescence, and lipid peroxidation of membranes and the possible contribution of these plant hormones in increasing the tolerance to this abiotic condition in lulo plants.

## 2 Materials and methods

### 2.1 Plant material and growing conditions

This experiment was performed in the greenhouses of the Universidad Nacional de Colombia, Bogotá campus (4°35′56´´N, 74°4′51´´W; 2556 masl) between August and December 2015 (16 weeks). Two-month-old lulo (*Solanum quitoense* cv. Septentrionale) seedlings purchased at a local nursery were transplanted into 3 L plastic pots (one plant per pot) containing a mixture of quartz sand and nutrient-free peat (Base 1 substrate, Klasman-Deilmann GmbH, Geeste, Germany) as low moisture retention substrate (1:1 v/v). The conditions of the greenhouse during the experiment were as follows: 22 ± 4 °C average temperature, relative humidity between 60 and 90%, and a day length of 12 h with a maximum photosynthetically active radiation (PAR) of 1500 mol m^−2^ s^−1^ recorded at noon. The seedlings underwent an acclimatization period of 30 days after transplantation.

During the experiment, plants were watered with a nutrient solution based on a complete liquid fertilizer (Nutriponic®; Walco SA, Colombia) at a dose of 5 mL·L^−1^ H_2_O. The nutrient solution concentration used was the following: 2.08 mM Ca (NO_3_)_2_·4H_2_O, 1.99 mM MgSO_4_·7 H_2_O, 2.00 mM NH_4_H_2_PO_4_, 10.09 mM KNO_3_, 46.26 nM H_3_BO_3_, 0.45 nM Na_2_MoO_4_·2H_2_O, 0.32 nM CuSO_4_·5H_2_O, 9.19 nM MnCl_2_·4H_2_O, 0.76 nM ZnSO_4_·7H_2_O, and 19.75 nM FeSO_4_·H_2_O. The irrigation volume of the nutrient solution was adjusted throughout the experiment depending on the pot capacity and plant growth to meet its water requirements. The following irrigation volume was used: 450 mL plant^−1^ week^−1^ from 1 to 30 days after transplanting (DAT), 600 mL plant^−1^ week^−1^ from 31 to 57 DAT, and 750 mL plant^−1^ week^−1^ from 58 to 80 DAT (end of the experiment). Plant irrigation requirements were estimated considering the plant's evapotranspiration demand (ETo) (quantified daily) using the gravimetric technique (Hainaut et al., 2016). Finally, the experiment lasted 80 days.

### 2.2 Drought stress treatments and foliar brassinosteroid analogue (DI-31) doses

After the acclimatization period (30 DAT), the lulo seedlings were divided into two groups of 25 plants each. The first group corresponded to seedlings under well-irrigated conditions, and the second consisted of water-stressed seedlings. Well-irrigated plants were watered up to 100% of their daily ETo needs for the duration of the experiment. On the other hand, water-stress treatment consisted of suppressing 100% of the daily ETo needs. Water-stressed plants were exposed to two water deficit periods (without water supply) for 7 consecutive days. Both water stress periods were carried out on the following dates: from 30 to 37 DAT (Period 1) and from 73 to 80 DAT (Period 2). Between stress periods, plants under water stress were subjected to a recovery time of 35 days, supplying 100% of their daily water needs.

Seedlings from both irrigation regimes (well-irrigated and water-stressed) were also subdivided into five other groups to establish foliar treatments with five different doses of a brassinosteroid analogue (DI-31). The doses were as follows: 0, 1, 2, 4, or 8 mL·L^−1^ of DI-31 [(25 R) – 3β. 5α – dihydroxy-spirostan-6-one] (Biomex DI-31®, Minerales exclusivos SA, Bogotá, Colombia). The concentrations of this analogue were selected based on the available literature on the use of brassinosteroid and analogues in plants under water stress conditions (Farooq et al., 2009; Yuan et al., 2010; Wang et al., 2015; Pérez-Borroto et al., 2021).

Foliar applications of DI-31 were performed at the beginning and during each period of water stress as well as in the recovery period. A total of six foliar sprays of the different DI-31 doses were carried out at 30, 33, 44, 60, 73, and 76 DAT. The application times were selected according to Fló rez – Velasco et al. (2015); these authors showed that multiple application sprays are appropriate to help lulo plants cope with abiotic stresses. Seedlings of the dose 0 mL·L^−1^ of DI-31 of both irrigation regimes (well-irrigated and water-stressed) were only sprayed with distilled water. Ten treatment groups were obtained at the end of the experiment: i) well-irrigated plants with foliar application of 1, 2, 4, or 8 mL·L^−1^ of DI-31; ii) well-irrigated plants without DI-31 application; iii) plants with water stress and foliar application of 1, 2, 4 or 8 mL·L^−1^ of DI-31, and iv) plants with water stress without DI-31 application. Finally, foliar applications of DI-31 were carried out between 07:00 and 09:00 h, using a Style 1.5 compression sprinkler (Matabi, Spain). The application volume of the sprinkler was 15 ml H_2_O per plant and the upper and lower surfaces of leaves were wetted until dripping. All foliar applications carried a surfactant adjuvant (INEX-A, Cosmoagro, Colombia) at a dose of 0.1% (v/v). A completely randomized factorial design (irrigation level vs. DI-31 doses) with five replicates was used to arrange the treatments, and each replicate consisted of a plant.

### 2.3 Leaf gas exchange parameters

One fully expanded leaf per plant from the upper part of the canopy was selected to carry out leaf gas exchange readings. The leaves were adapted to the surrounding environmental conditions without touching their surface to prevent stomatal closure before measurements. Stomatal conductance (g_*s*_) readings were taken over two days before the end of the experiment (79 and 80 DAT) using a portable porometer (SC-1, Decagon Devices Inc., Pullman, WA, US). Net photosynthesis (P_*N*_) was estimated using a portable meter (LiCOR 6200, Lincoln, Nebraska, USA). One set of 2-d measurements (79 and 80 DAT) was carried out to determine P_*N*_ and g_*s*_ between 1100 and 1200 h following the methodology described by Lombardini et al. (2009). The conditions within the LiCOR chamber during P_*N*_ readings were the following: leaf temperature of 25 ± 5 °C, PAR ≥800 μmol m^−2^∙s^−1^, and leaf-to-air vapor pressure difference of 1.8 ± 0.5 kPa. Intrinsic water use efficiency (WUE_*i*_) was determined as the P_*N*_ to g_*s*_ ratio (Flexas et al., 2001). Finally, the gravimetric technique described by Sá nchez – Reinoso et al. (2014) was used to obtain the total plant transpiration (*E*). Pots were covered with plastic wrap to prevent water loss by evaporation and weighted daily before and after irrigation during the second water stress period (73–80 DAT). The transpiration rate was expressed in mg H_2_Og^−1^FW∙h^−1^.

### 2.4 Chlorophyll fluorescence parameters

Chlorophyll *a* fluorescence parameters were determined on the same leaves used to estimate gas exchange parameters (P_*N*_ and g_*s*_) utilizing a modulated fluorometer (MINI-PAM, Walz, Effeltrich, Germany). The leaves were previously adapted to the dark using clips for 20 min. After dark adaptation, we calculated the maximum quantum efficiency of PSII (F_v_/F_m_), photochemical quenching (qP), and non-photochemical quenching (NPQ) using an actinic light pulse of up to 2,600 μmol m^−2^∙s^−1^ on the adaxial leaf surface to determine fluorescence parameters. These readings were taken at 80 DAT.

### 2.5 Screening of water stress tolerance in terms of F_v_/F_m_ ratio

The decrease in the maximum quantum efficiency of PSII (DQE) under water stress was calculated using the F_v_/F_m_ ratio readings obtained at the end of the experiment (80 DAT). compared to foliar doses of DI-31. DQE was estimated using Eq. (1) described by Chá ves – G ó mez et al., 2020.

(1) DQE=[(Fv/FmWI−(Fv/FmWS)/(Fv/FmWI))]×100

where WI represents well-irrigated plants without foliar applications of DI-31, and WS represents plants subjected to water stress and with foliar applications of DI-31 at the evaluated doses (0, 1, 2, 4, and 8 mL·L^−1^ of DI-31).

Subsequently, foliar treatments with different DI-31 doses were classified into the following four categories: high tolerance: DQE ≤25; moderate tolerance: between 26 and 41; low tolerance: between 42 and 55; susceptible ≥56.

### 2.6 Leaf photosynthetic pigments

The equations by Wellburn (1994) were used to determine the contents of total chlorophyll (TChl) and carotenoids (Cx + c) in leaves at 80 DAT. Leaf tissue samples of approximately 30 mg were collected from the same leaves used to estimate P_*N*_ and g_*s*_. Samples were homogenized in 3 mL of 80% acetone (v/v) and then centrifuged (Model 420101, Becton Dickinson Primary Care Diagnostics, MD, USA) at 5000 rpm for 10 min to eliminate particles. Acetone was added to dilute the supernatant to a final volume of 6 mL (Sims and Gamon, 2002). A spectrophotometer (Spectronic BioMate 3 UV-vis Thermo, Madison, WI) was used to determine the content of TChl at 663 and 646 nm, and Cx + c at 470 nm.

### 2.7 Production of malondialdehyde (MDA)

Malondialdehyde (MDA) production (lipid peroxidation of membranes) was estimated using the thiobarbituric acid (TBA) method by Hodges et al. (1999). Approximately 300 mg of plant material from the same leaves used to measure gas exchange parameters was homogenized and stored in liquid nitrogen. Subsequently, the samples were centrifuged (Model 420101, Becton Dickinson Primary Care Diagnostics, MD, USA) at 5000 rpm for 10 min, and a spectrophotometer (Spectronic BioMate3UV-Vis, Thermo, Madison, WI) was used to estimate absorbances at 440, 532, and 600 nm. The extinction coefficient (157 M mL^−1^) was considered to determine the MDA concentration, and MDA readings were also taken at 80 DAT.

### 2.8 Leaf relative water content

Fully expanded leaves were collected from the middle portion of the canopy to determine the relative water content (RWC) also at 80 DAT. After determining the fresh weights (FW), the leaves were put in distilled water at 4 °C for 24 h in a dark room to determine the turgid weight (TW). The dry weight (DW) was calculated by heating turgid leaves in an oven at 70 °C for 48 h. The RWC was estimated according to Smart and Bingham (1974), using Eq. (2):

(2) $$RWC=\left(\frac{FW-DW}{TW-DW}\right)\times 100$$

### 2.9 Plant growth parameters

Plant height was recorded at the end of the experiment (80 DAT) using a ruler. Lulo plants were then harvested, and their biomass was separated into each of the organs (leaves, stems, and roots). Photographs of all the leaves in the plant canopy (D3300, Nikon, Thailand) were taken to determine the leaf area (LA) of each plant. All photographs were saved as TIFF (Tagged Image File Format) images and then processed using Java software (Image J; National Institute of Mental Health, Bethesda, MD) to calculate LA. Plant organs were dried in an oven at 70 °C for 72 h until constant weight to estimate the dry weights of leaves (LDW), stems, roots, and total (TDW); additionally, the dry matter (DM) partitioning was determined. Finally, the following indices were obtained: specific leaf area (SLA = LA/LWD; c m^2^·g^−1^) (Gunn et al., 1999), leaf mass per area (LMA = LDW/LA; mg·cm^2^) (Cheng et al., 2014) and equivalent water thickness (EWT = (leaf fresh weight (LFW) – LDW)/LA; mg·cm^2^) (Yilmaz et al., 2008).

### 2.10 Relative tolerance index (RTI)

The effect of foliar applications of DI-31 on the tolerance of lulo plants to water stress conditions was determined using the relative tolerance index (RTI). RTI was estimated using the values of net photosynthesis (P_*N*_) of the different treatments with DI-31 under drought stress conditions in relation to the treatment under well-irrigated conditions and without DI-31 applications. The RTI was calculated at 80 DAT, using Eq. (3) from Chá vez – Arias et al. (2020) with some modifications:

(3) $$RTI=\left(\frac{P_{N}\;under\;water\;stress\;conditions}{P_{N}\;under\;well-irrigated\;conditions}\right)\times 100$$

### 2.11 Experimental design and statistical analysis of data

For data analysis, a factorial arrangement was used where the plant water status (well-irrigated plants vs. water-stressed plants) was the first factor and the DI-31 doses (0, 1, 2, 4, or 8 mL·L^−1^) corresponded to the second factor. Five plants were used per treatment. We performed an analysis of variance (ANOVA) and a post hoc Tukey test for comparison of means when significant differences (P ≤ 0.05) were observed. A correlation analysis between RTI and TDW or MDA was conducted to find the best treatments under water deficit conditions (water-stressed plants). The arcsine function was used to transform percentage values. Data were processed using the software Statistix v 9.0 (Analytical Software, Tallahassee, FL.). All figures and the three-dimensional graph were prepared using SigmaPlot (version 12.0; Systat Software, San José, CA, USA), and the correlation analysis was conducted by the same software.

## 3 Results

### 3.1 Leaf growth parameters and relative water content (RWC)

**Figure 1:**
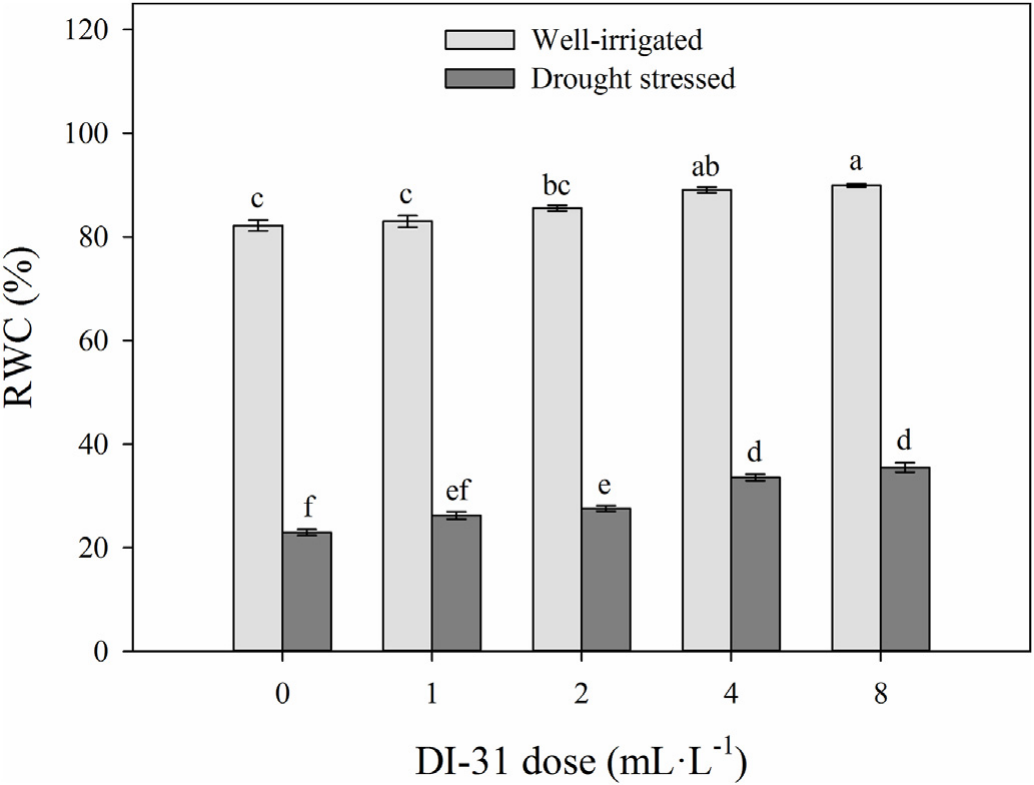
Relative water content (RWC) in well-irrigated (light grey bars) and drought-stressed (dark gray bars) lulo (*Solanum quitoense* Lam.) plants sprayed with 1, 2, 4 and 8 mL·L^−1^ of brassinosteroid analogue (DI-31) at 80 days after transplanting (DAT). Each column represents the mean of five data ± standard error (*n* = 5). Bars followed by different letters indicate statistically significant differences according to the Tukey test (*p* ≤ 0.05).

Significant differences were observed in the interaction between irrigation regimes and doses of brassinosteroid analogue on the relative water content (RWC) (*p* = 0.02) and leaf growth parameters such as leaf area (LA) (*p* = 0.000), leaf dry weight (LDW) (*p* = 0.014), specific leaf area (SLA) (*p* = 0.033), leaf mass per area (LMA) (*p* = 0.019) and equivalent water thickness (EWT) (*p* = 0.000) at 80 DAT. The RWC increased significantly with foliar application of DI-31 in plants under both irrigation regimes. In the group of well-irrigated plants, foliar applications with 4 and 8 mL·L^−1^ of DI-31 increased RWC by 9% (mean values of 89.5%) compared to plants without DI-31 application (82.2%). A much higher increment in RWC was observed in drought-stressed plants. RWC increased significantly by 20.2, 46.3, and 54.7% after exogenous application of 2, 4, and 8 mL·L^−1^ of DI-31, respectively, compared to the treatment with 0 mL·L^−1^ of DI-31 in the same group of plants (22.9%) (Figure 1).

LA, LDW, SLA, and EWT were also highly affected by the drought stress condition, registering the lowest values in plants without foliar application of DI-31 (Table 1). A positive response was observed in plants under both irrigation regimes when sprayed with DI-31. Foliar applications with 8 mL·L^−1^ of DI-31 caused the greatest increases of 30.1% in LA, 25.8% in LDW, 19.8% in SLA, and 18.8% in EWT compared to the other treatments in drought-stressed plants. In contrast, LMA showed an inverse behavior since the highest values of this variable were observed in drought-stressed plants without exogenous application of DI-31 (12.1 mg cm^2^). A much lower LMA value was recorded in all the doses of DI-31 used (1, 2, 4, and 8 mL·L^−1^) registering mean values of 11.6 mg cm^2^ in lulo plants subjected to water deficit stress. The highest values of LA, LDW, and SLA in well-irrigated plants were recorded in foliar treatments with 4 and 8 mL·L^−1^ of DI-31. All DI-31 doses did not show any effect on the variables LMA and EWT in this group of plants (Table 1).

**Table 1:** Leaf area (LA), leaf dry weight (LDW), specific leaf area (SLA), leaf mass per area LMA) and equivalent water thickness (EWT) of lulo (*Solanum quitoense* Lam.) plants exposed to drought and sprayed with 1, 2, 4 and 8 mL·L^−1^ of brassinosteroid analogue (DI-31) at 80 days after transplanting (DAT).

Irrigation treatment	DI-31 dose (mL·L^−1^)	LA	LDW	SLA	LMA	EWT
		(cm^2^)	(g dry matter)	(cm^2^·g^−1^)	(mg·cm^−2^)	(mg·cm^−2^)
Well-irrigated	0	1353.7 c^a^	11.1 b	123.4 c	8.2 c	42.7 a
	1	1389.6 bc	11.4 b	123.9 c	8.1 c	43.6 a
	2	1464.4 b	11.6 b	126.9 bc	7.9 c	43.9 a
	4	1612.7 a	12.2 ab	132.7 ab	7.8 c	44.3 a
	8	1679.3 a	13.3 a	134.1 a	7.6 c	44.6 a
Drought-stressed	0	524.5 e	6.3 d	72.3 e	12.8 a	31.1 d
	1	554.3 e	7.3 cd	76.3 e	12.1 b	34.4 c
	2	663.2 d	7.7 c	85.7 d	11.7 b	35.3 bc
	4	672.3 d	7.8 c	86.6 d	11.5 b	36.3 bc
	8	682.3 d	7.9 c	86.9 d	11.4 b	36.6 b
Significance *(p* value*)*		0.0000	0.0143	0.0193	0.0333	0.0005
CV (%)^b^		3.45	5.33	3.41	3.18	2.42

a Values *(n* = 5) within the same column and followed by different letters are significantly different according to the Tukey test (*p* ≤ 0.05).

b CV: Coefficient of variation.

**Figure 2:**
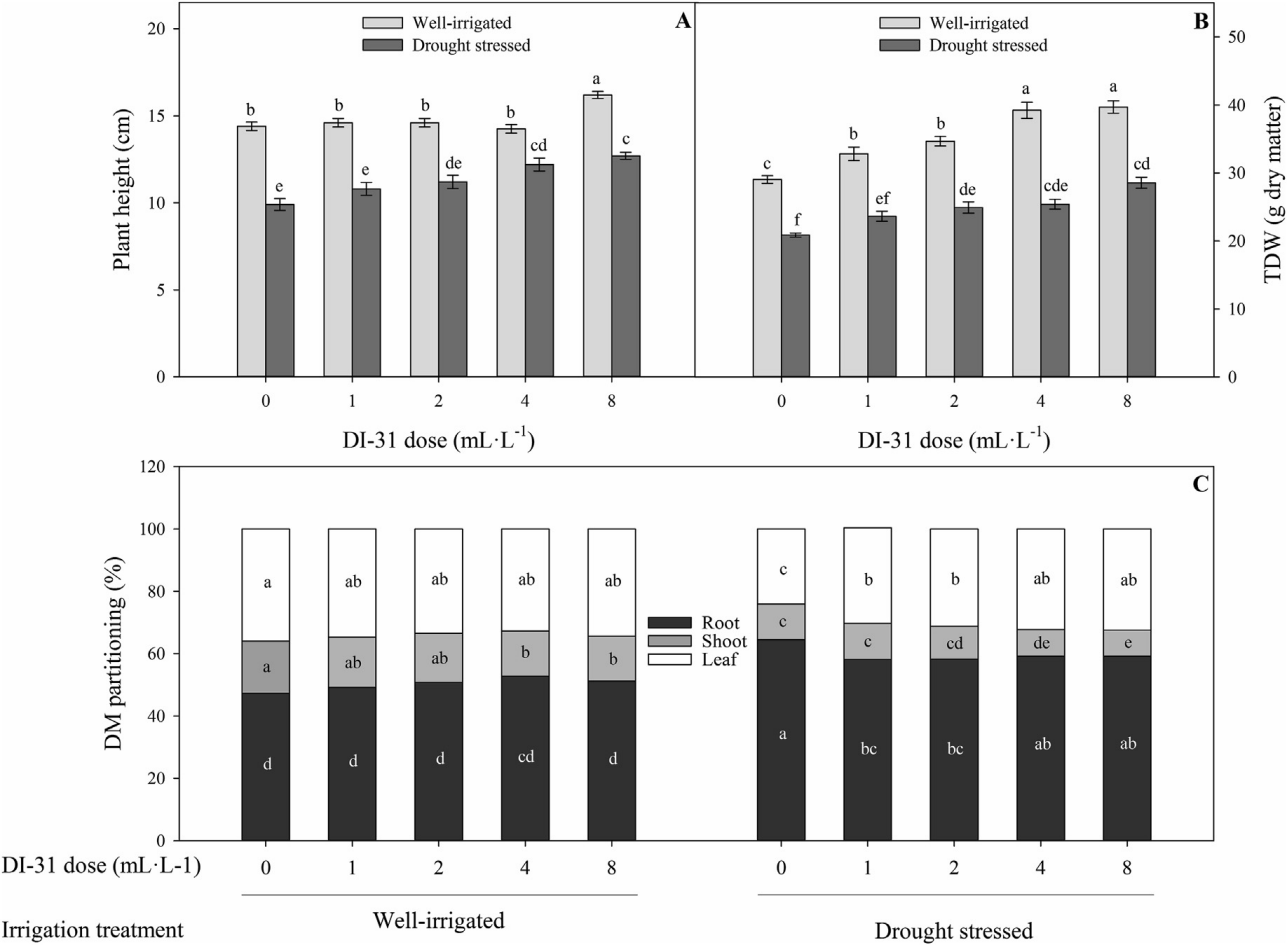
Plant height (A), total dry weight (TDW) (B) and dry matter (DM) partitioning (C) in well-irrigated (light grey bars) and drought-stressed (dark gray bars) lulo (*Solanum quitoense* Lam.) plants sprayed with 1, 2, 4 and 8 mL·L^−1^ of brassinosteroid analogue (DI-31) at 80 days after transplanting (DAT). Each bar chart summarizes the mean of five data ± standard error (*n* = 5). Bars followed by different letters indicate statistically significant differences according to the Tukey test (*p* ≤ 0.05).

Plant growth parameters such as plant height (*p* = 0.02), total dry weight (TDW) (*p* = 0.011), and dry matter (DM) partitioning were also affected by the interaction between irrigation regimes and doses of DI-31 at 80 DAT (Figure 2). In treatments with well-irrigated plants, the application of 8 mL·L^−1^ of DI-31 significantly increased the plant height (16.2 cm) compared to the other treatments (0, 1, 2, and 4 mL·L^−1^ of DI-31), registering mean values of 14.4 cm (Figure 2A). A progressive increase in TDW was also observed with the increment in the foliar dose of DI-31. The highest values of TDW were recorded in the treatments with 4 and 8 mL·L^−1^ of DI-31 (∼39.4 g DM) compared to plants without DI-31 application (29.1 g DM) (Figure 2B). The foliar application of DI-31 also generated a positive effect on plant height and TDW in plants subjected to drought stress. These growth parameters were favored mainly by the foliar application of 4 (12.2 cm for plant height and 25.4 g DM for TDW) and 8 mL·L^−1^ (12.7 cm for plant height and 28.5 g DM for TDW) of DI-31 in plants under drought conditions compared to plants without exogenous application of DI-31 (9.9 cm for plant height and 20.9 g DM for TDW) (Figure 2A and B).

Foliar applications of DI-31 did not alter the DM partitioning in leaves and roots of well-irrigated plants. However, the application of 4 and 8 mL·L^−1^ of DI-31 generated a lower percentage of DM accumulation in the stem (∼14.4%) compared to the other treatments under well-irrigated conditions (∼16.2%). Under conditions of drought stress, the foliar application of this analogue showed two different effects on the DM partitioning of lulo plants: the first effect was an increase in the percentage of leaf DM with the application of mainly 4 and 8 mL·L^−1^ of DI-31, registering mean values of 32.3% compared to untreated plants (24.1%). The second effect was a decrease in the percentage of stem DM with the exogenous application of 4 and 8 mL·L^−1^ of DI-31 since this group of plants showed mean values of 8.4% compared to plants without foliar application of DI-31 (11.4%). A reduction in the percentage of root DM was also recorded after the exogenous application of 1, 2, 4, or 8 mL·L^−1^ of DI-31, registering mean values of 58.6% compared to treatment plants with 0 mL·L^−1^ of DI-31 (64.4%) (Figure 2C).

### 3.2 Leaf gas exchange parameters

**Figure 3:**
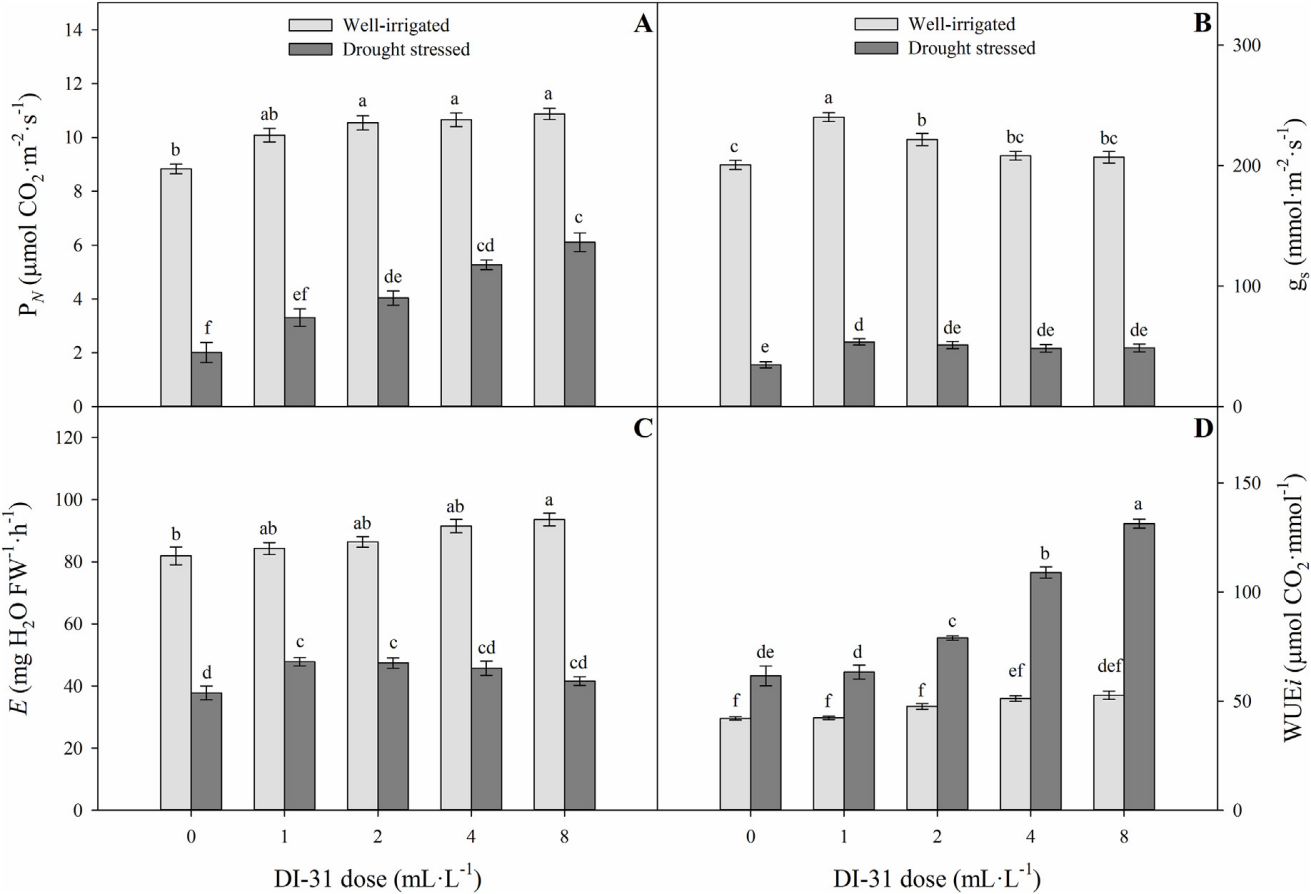
Net photosynthetic rate (P_*N*_) (A), stomatal conductance (g_*s*_) (B), transpiration rate (*E*) (C) and intrinsic water-use efficiency (WUE_*i*_) (D) in well-irrigated (light grey bars) and drought-stressed (dark gray bars) lulo (*Solanum quitoense* Lam.) plants sprayed with 1, 2, 4 and 8 mL·L^−1^ of brassinosteroid analogue (DI-31) at 80 days after transplanting (DAT). Each bar chart summarizes the mean of five data ± standard error (*n* = 5). Bars followed by different letters indicate statistically significant differences according to the Tukey test (*p* ≤ 0.05).

Significant differences were also observed on leaf gas exchange parameters (P_*N*_, g_*s*_, *E,* and WUE_*i*_) of lulo plants for the interaction between irrigation regimes and foliar doses of BRs analogue (DI-31) at 80 DAT (Figure 3). In drought-stressed plants, foliar application of DI-31 at the doses evaluated (1, 2, 4, and 8 mL·L^−1^ of DI-31) showed two responses on gas exchange parameters. In the first case, P_*N*_ and WUE_*i*_ showed increased values with the increment in the foliar dose of DI-31, obtaining the highest values with the treatment of 8 mL·L^−1^ of DI-31 (6.1 μmol CO_2_ m^−2^·s^−1^ for P_*N*_ and 131.3 μmol CO_2_·mmol H_2_O^−1^ for WUE_*i*_) compared to untreated plants (2.01 μmol CO_2_ m^−2^·s^−1^ for P_*N*_ and 61.5 μmol CO_2_·mmol H_2_O^−1^ for WUE_*i*_) (Figure 3A and D). In the second case, plants with foliar application of 1 mL·L^−1^ of DI-31 enhanced acclimatization expressed as the highest g_*s*_ values (53.6 mmol CO_2_ m^−2^·s^−1^) and *E* (47.8 mg H_2_O FW^−1^·h^−1^) compared to untreated lulo plants (34.6 mmol CO_2_ m^−2^·s^−1^ for g_*s*_ and 37.8 mg H_2_O FW^−1^·h^−1^ for *E*) (Figure 3B and C).

Foliar applications of DI-31 positively affected the gas exchange parameters (P_*N*_, g_*s*_, *E,* and WUE_*i*_) of well-irrigated plants. Increased values of P_*N*_ were observed with exogenous applications of 2, 4, and 8 mL·L^−1^ of DI-31, obtaining mean values of 10.7 μmol CO_2_ m^−2^·s^−1^ compared to untreated plants (8.8 μmol CO_2_ m^−2^·s^−1^) (Figure 3A). Higher values of g_*s*_ were also recorded with the foliar application of 1 and 2 mL·L^−1^ of DI-31 (∼230.9 mmol CO_2_ m^−2^·s^−1^) compared to plants without application of plant hormones (200.5 mmol CO_2_ m^−2^·s^−1^) (Figure 3B). On the other hand, *E* values of lulo plants under well-irrigated conditions significantly increased by 14.3% compared to the treatment with 0 mL·L^−1^ of DI-31 (81.9 mg H_2_O FW^−1^·h^−1^) after foliar spray with 8 mL·L^−1^ of DI-31 (Figure 3C). WUE_*i*_ values did not significantly differ between treatments with various concentrations of DI-31 in well-irrigated plants (Figure 3D).

### 3.3 Chlorophyll fluorescence parameters

**Figure 4:**
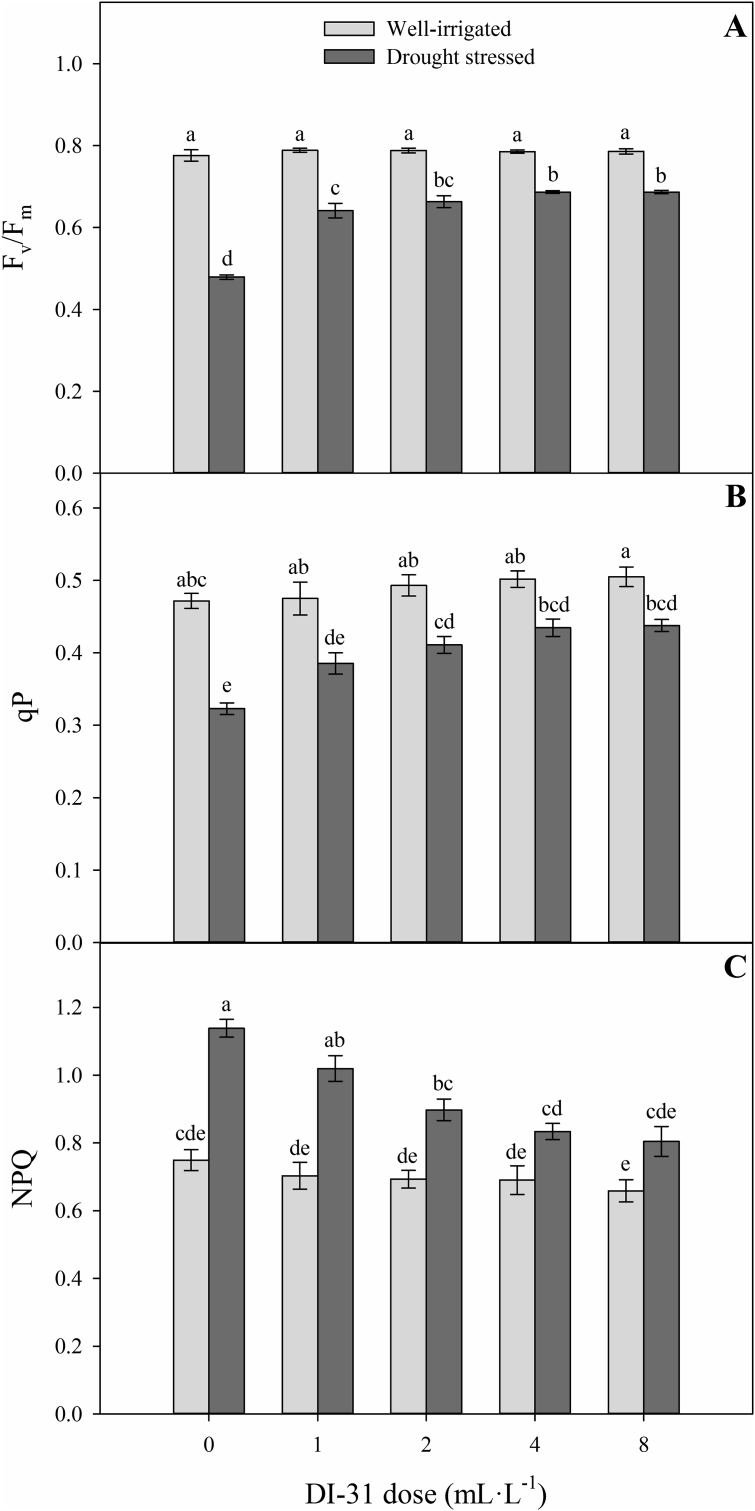
Maximum quantum efficiency of PSII (F_v_/F_m_) (A), photochemical quenching (qP) (B) and non-photochemical quenching (NPQ) (C) in well-irrigated (light grey bars) and drought-stressed (dark gray bars) lulo (*Solanum quitoense* Lam.) plants sprayed with 1, 2, 4 and 8 mL·L^−1^ of brassinosteroid analogue (DI-31) at 80 days after transplanting (DAT). Each bar chart summarizes the mean of five data ± standard error (*n* = 5). Bars followed by different letters indicate statistically significant differences according to the Tukey test (*p* ≤ 0.05).

Figure 4 shows the chlorophyll fluorescence parameters evaluated in lulo plants at 80 DAT. Significant differences were also found on the F_v_/F_m_ ratio (*p* = 0.000), qP (*p* = 0.025) and NPQ (*p* = 0.002) for the interaction between irrigation regimes and foliar doses of DI-31. Plants of the treatments under well-irrigated conditions did not show differences in chlorophyll fluorescence parameters between foliar doses of DI-31 (0, 1, 2, 4, and 8 mL·L^−1^ of DI-31) (Figure 4). Increased F_v_/F_m_ ratio and qP values were observed with higher doses of DI-31 under conditions of water deficit. The highest values of these variables were registered in plants with foliar sprays of 4 and 8 mL·L^−1^ of DI-31 (0.69 for F_v_/F_m_ and 0.44 for qP) compared to plants of the 0 mL·L^−1^ treatment (0.48 for F_v_/F_m_ and 0.32 for qP) (Figure 4A and B). In contrast, the values of NPQ showed a drop with increasing foliar doses of DI-31. The lowest values of this variable were obtained in the treatments of 4 and 8 mL·L^−1^ of this plant hormone (∼0.81) compared to plants without foliar application of DI-31 (1.14) (Figure 4C).

### 3.4 Photosynthetic pigment content and malondialdehyde (MDA) production

**Figure 5:**
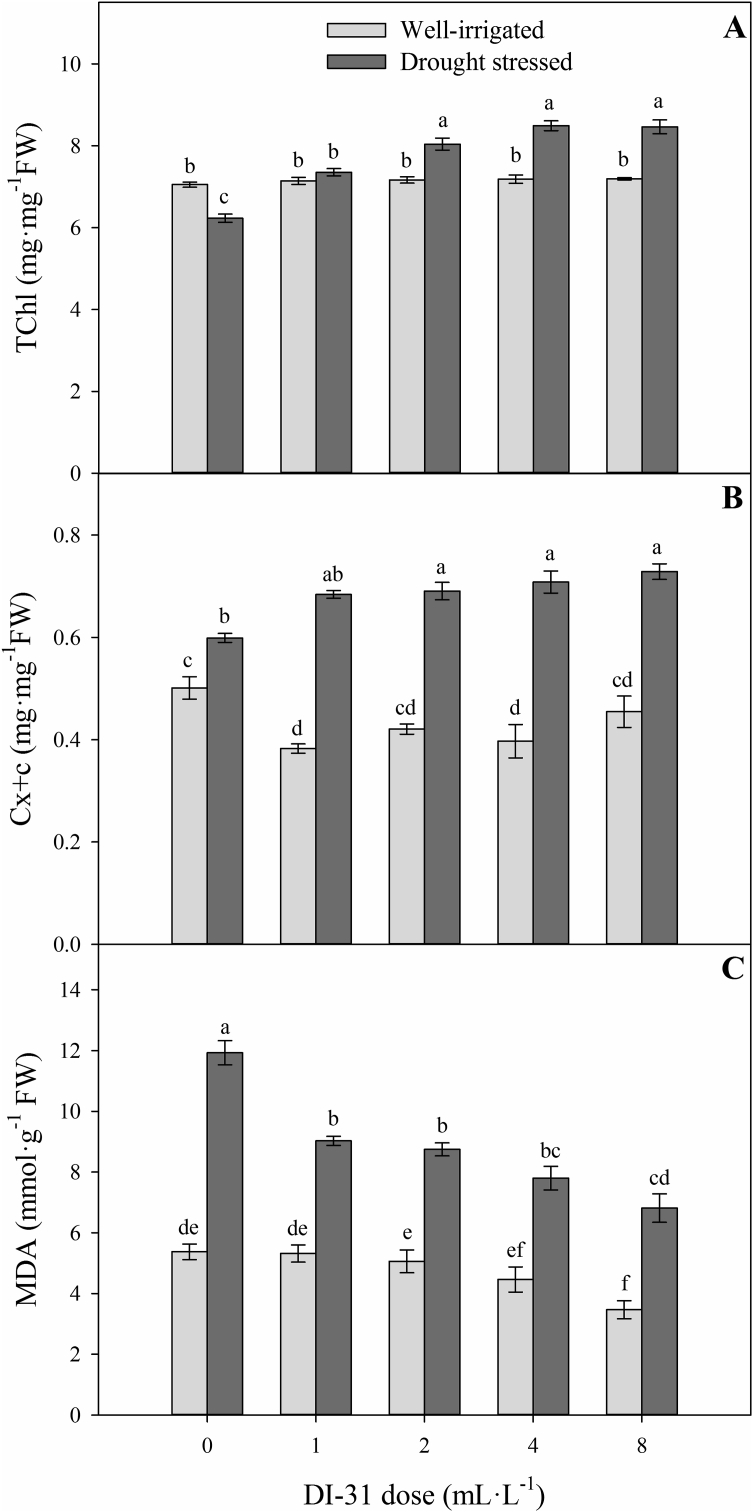
Total chlorophyll content (TChl) (A), carotenoids (Cx + c) (B) and malondialdehyde production (MDA) (C) in well-irrigated (light grey bars) and drought-stressed (dark gray bars) lulo (*Solanum quitoense* Lam.) plants sprayed with 1, 2, 4 and 8 mL·L^−1^ of brassinosteroid analogue (DI-31) at 80 days after transplanting (DAT). Each bar chart summarizes the mean of five data ± standard error (*n* = 5). Bars followed by different letters indicate statistically significant differences according to the Tukey test (*p* ≤ 0.05).

The contents of total chlorophyll (TChl) and carotenoids (Cx + c) were also affected by the interaction of irrigation regimes and foliar doses of BRs analogue (*p* = 0.000 and *p* = 0.000, respectively) at 80 DAT (Figure 5). Foliar application of DI-31 at the doses evaluated did not show differences in TChl in well-irrigated lulo plants. However, Cx + c values decreased with the application of treatments with 1 and 4 mL·L^−1^ of DI-31 (0.38 mg mg^−1^ FW and 0.4 mg mg^−1^ FW, respectively) compared to the treatment of 0 mL·L^−1^ of DI-31 (0.5 mg mg^−1^ FW) in the same group of plants (Figure 5A and B). When plants were under drought stress conditions, the foliar application of 2, 4, and 8 mL·L^−1^ of DI-31 showed a positive effect on photosynthetic pigments (∼8.3 mg mg^−1^ FW for TChl and ∼0.71 mg mg^−1^ FW for Cx + c) compared to plants without foliar treatment with DI-31 (6.2 mg mg^−1^ FW for TChl and of 0.6 mg mg^−1^ FW for Cx + x) (Figure 5A and B).

Statistical differences (*p* = 0.0001) were also found for MDA production in the interaction between irrigation regimes and foliar doses of DI-31 at 80 DAT (Figure 5C). In general, a progressive decrease in MDA content was recorded with increasing doses of DI-31 in both irrigation groups. In well-irrigated lulo plants, MDA content was lower in the treatment with the exogenous application of 8 mL·L^−1^ of DI-31 (3.5 mmol g^−1^ FW) compared to plants without any DI-31 application (5.4 mmol g^−1^ FW). Exposure to drought conditions generated the highest values of MDA production (11.9 mmol g^−1^ FW) in lulo plants without foliar treatment. However, the application of the doses evaluated registered a drop in the values of this variable, obtaining lower lipid peroxidation (MDA) with the foliar application of 8 mL·L^−1^ of DI-31 (6.8 mmol g^−1^ FW).

### *3.5* *Detection of tolerance to water stress promoted by foliar application of brassinosteroid analogue* DI-31

**Figure 6:**
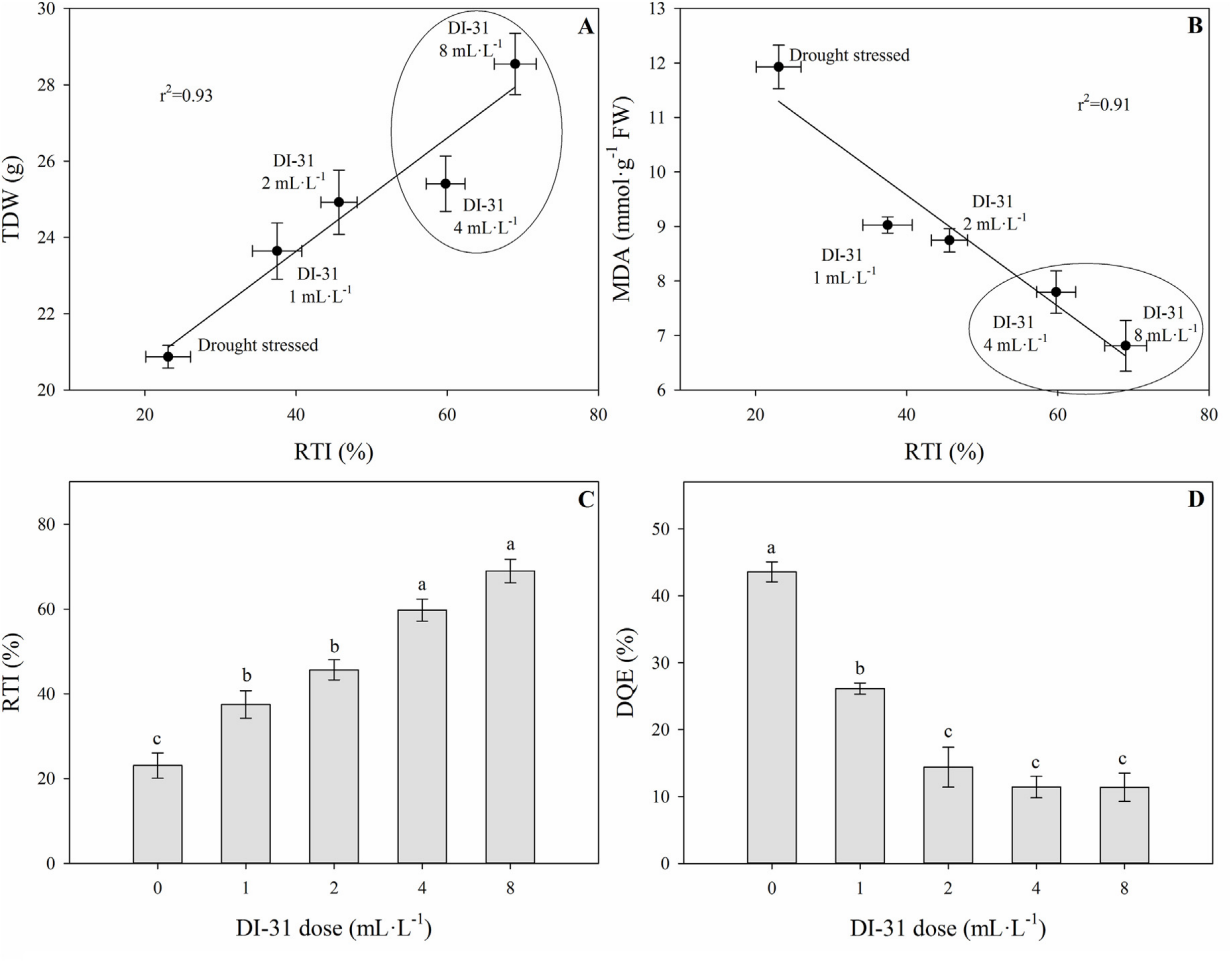
Correlation between the total dry weight (TDW) (A) or malondialdehyde production (MDA) (B) and the relative tolerance index (RTI) (C), and the decrease in the maximum quantum efficiency of PSII (DQE) (D) in lulo (*Solanum quitoense* Lam.) plants exposed to drought and sprayed with 1, 2, 4 and 8 mL·L^−1^ of brassinosteroid analogue (DI-31) at 80 days after transplanting (DAT). Bars and points represent the mean of five data ± standard error (*n* = 5). Bars followed by different letters indicate statistically significant differences according to the Tukey test (*p* ≤ 0.05).

**Figure 7:**
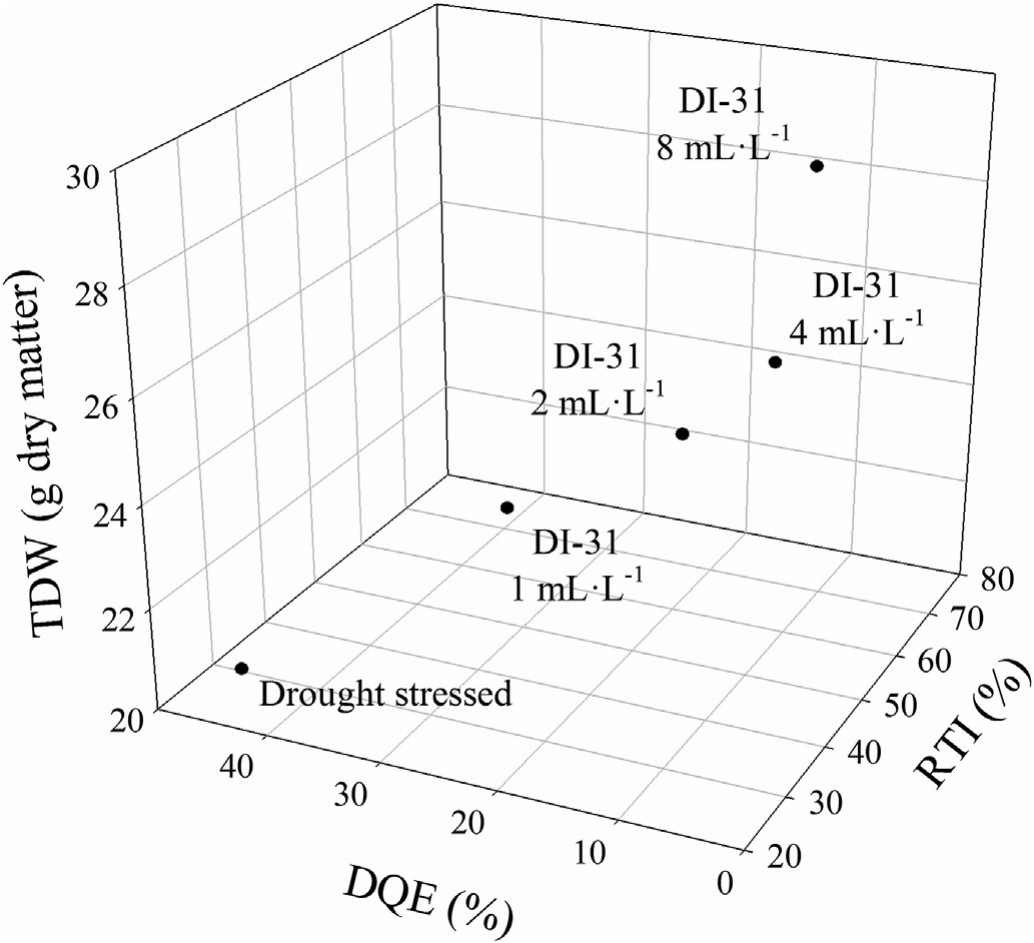
Three-dimensional plot (total dry weight (TDW), decrease in the maximum efficiency of PSII (DQE) and relative tolerance index (RTI)) for lulo (*Solanum quitoense* Lam.) plants exposed to drought and sprayed with 1, 2, 4 and 8 mL·L^−1^ of brassinosteroid analogue (DI-31) at 80 days after transplanting (DAT). Data represents the mean of five data ± standard error (*n* = 5).

The relative tolerance index (RTI), correlations between RTI and TDW or MDA, and the decrease in the maximum quantum efficiency of PSII (DQE) were determined at 80 DAT to identify the foliar doses of DI-31 that could be used as a possible strategy for the management of drought stress in lulo plants (Figure 6). The RTI and its correlations with TDW (r^2^ = 0.93) and MDA production (r^2^ = 0.91) showed that the treatments with foliar applications of 4 and 8 mL·L^−1^ of DI-31 exhibited a higher accumulation of dry matter in the entire plant and lower lipid peroxidation of membranes in drought-stressed plants (Figure 6A and B). This was reflected in a higher RTI for the dose of 8 mL·L^−1^ of DI-31 (69%) followed by the dose of 4 mL·L^−1^ of DI-31 (59.8%) in lulo plants under water deficit conditions (Figure 6C). DQE confirmed all the previous observations; this index showed that drought stress had a lesser effect on the quantum efficiency of PSII in lulo plants treated with 2 (14.4%), 4 (11.4%), and 8 (11.3%) mL·L^−1^ of DI-31 with values lower than 25%. As a result, these plants were classified with a good level of tolerance to conditions of water stress (Figure 6D and Table 2). Finally, the three-dimensional graph (TDW, RTI, and DQE) also confirmed the results of the correlation analysis where lulo plants treated with exogenous applications of 4 and 8 mL·L^−1^ of DI-31 showed enhanced physiological behavior (lower affectation of P_*N*_, F_v_/F_m_ ratio, and photosynthetic pigments and a lower MDA production) under conditions of drought stress (Figure 7).

**Table 2:** Classification of treatments with foliar applications of brassinosteroid analogue (DI-31) based on the decrease in the maximum quantum efficiency of photosystem II (Fv/Fm ratio) (DQE) in lulo (*Solanum quitoense* Lam.) plants under drought stress at 80 days after transplanting (DAT).

DQE (%)^a^	Classification	Treatments studied
DQE ≤25	Good tolerance	DI-31 2 mL·L^−1^, DI-31 4 mL·L^−1^, DI-31 8 mL·L^−1^
26 ≥ DQE ≤41	Moderate tolerance	DI-31 1 mL·L^−1^
42 ≥ DQE ≤55	Low tolerance	Drought-stressed plants
DQE ≥56	Very low tolerance	-

a DQE - decrease in the maximum quantum efficiency of photosystem II (F_v_/F_m_ ratio) in lulo plants exposed to drought and sprayed with DI-31 compared to untreated well-irrigated plants.

## 4 Discussion

The results of this research indicated that water deficit stress (irrigation suspension) caused significant damage to lulo plants. Drought conditions caused a negative impact on plant water status (lower RWC), plant growth, and photosynthetic function (lower P_*N*_, g_*s*_, F_v_/F_m,_ and TChl) and promoted a higher lipid peroxidation (higher MDA content). Leaf RWC is considered one of the most appropriate indicators to estimate the water status of plants exposed to water stress conditions (Jungklang et al., 2017). In this study, RWC was reduced because of water stress in lulo plants treated only with distilled water, confirming that this group of plants was subjected to stressful conditions. Similar results were observed by Jangid and Dwivedi (2017) in tomato (*Solanum lycopersicum* L.) plants and Plazas et al. (2019) in eggplant (*Solanum melongena* L.) plants registering a reduction in RWC of 27.6 and 35%, respectively, under conditions of water deficit stress. In this study, a significant increase in RWC was observed at higher doses of DI-31 (4 or 8 mL·L^−1^) under drought stress. Pérez-Borroto et al. (2021) also reported that foliar applications of DI-31 increased RWC by 16% in *Arabidopsis thaliana* L. plants under drought. A higher RWC in lulo plants under conditions of water stress may be associated with the fact that DI-31 is involved in the plant's osmotic adjustment process, promoting soluble carbohydrate accumulation (starch and sucrose), and the development of primary and lateral roots in plants under drought stress conditions (Khamsuk et al., 2018; Pérez-Borroto et al., 2021).

Low water availability reduced growth parameters such as LA, LDW, TDW, plant height, and DM partitioning of lulo plants (Table 1 and Figure 2). The reduction in growth caused by drought has also been described in other Solanaceae species such as potato (*Solanum tuberosum* L.) (Banik et al., 2016; Hill et al., 2021), tomato (Shi et al., 2016), and Russian box thorn (*Lycium ruthenicum* Murr.) (Guo et al., 2016). However, the exogenous application of DI-31 resulted, in general, in increased shoot growth in lulo plants exposed to water deficit. These responses may be related to the beneficial effects of analogue BRs (DI-31) on gas exchange parameters including a higher P_*N*_ (Figure 3). The increase in P_*N*_ may be mediated by the analogue's effect to promote a higher photosynthetic area and growth rate, and adequate stomatal regulation. This BRs analogue also plays an important role in the process of cell division in an environment of abiotic stress (Lima and Lobato, 2017; Tanveer et al., 2019; Pérez-Borroto et al., 2021). Serna et al. (2012a) also described positive results of DI-31 applications on growth parameters such as fresh weight and yield of endive (*Cichorium endivia* L.) plants. Finally, lower dry matter partitioning to roots was observed under water stress conditions despite the application of the analogue. This allows us to infer that root growth may have been inhibited by the abiotic stress rather than by the use of the analogue. This statement is based on the DM partitioning in well-irrigated plants. Hola (2019) reports that the lack of response may be due to the fact that the exogenous application of BRs or the analogue is limited locally to the site of application and is not transported to other organs.

Drought stress reduced leaf gas exchange proprieties (P_*N*_, g_*s,*_ and *E*) in lulo plants. Liang et al. (2020) observed that drought stress also reduced P_*N*_, g_*s,*_ and *E* in tomato plants. However, foliar DI-31 sprays improved leaf P_*N*_ of lulo plants under stress conditions. An increase in the values of P_*N*_ was also recorded in lettuce (*Lactuca sativa* L.) plants after the exogenous application of DI-31 (Serna et al., 2012b). It has been reported that BRs analogues such as DI-31 promote photosynthetic capacity through the upregulation of Rubisco (the enzyme in charge of intercellular CO_2_ assimilation) and chlorophyllase, and the transcription of encoded genes involved in photosynthesis under stress conditions (Anwar et al., 2018; Hussain et al., 2020; Pérez-Borroto et al., 2021). On the other hand, foliar sprays of analogue BRs (DI-31) at low concentrations can induce stomatal opening under stress conditions, improving leaf gas exchange properties and plant water status (Serna et al., 2012c; Chá vez – Arias et al., 2020). In this study, the lower foliar concentrations of DI-31 (1 or 2 mL·L-1) caused higher g_*s*_ values related to the increase in *E* (Figure 3B and C). Likewise, foliar applications also increased the WUE_*i*_ values in lulo plants under stress. Pereira et al. (2019) obtained an increment in WUE_*i*_ of more than 250% caused by the application of 50 and 100 nM of BRs in soybean (*Glycine max* L.) plants subjected to water deficit stress compared to untreated plants. BRs can enhance water relations through their participation in the modification and/or manipulation of the structure and stability of the plasma membrane under stress conditions (Hayat et al., 2010; Ramakrishna and Rao, 2015).

Drought stress altered leaf chlorophyll *a* parameters (a high NPQ, and low F_v_/F_m_ and qP). The determination of chlorophyll *a* fluorescence parameters is a rapid and non-destructive tool used to study the tolerance or level of acclimatization of plants to environmental stress (Thussagunpani et al., 2015; Chá vez – Arias et al., 2019). Raja et al. (2020) also recorded lower F_v_/F_m_ and qP, and higher NPQ values of tomato plants exposed to water stress. In contrast, the foliar DI-31 application increased the values of F_v_/F_m_ and qP and caused a decrease in NPQ in this experiment. BRs have been reported to alleviate the photoinhibition promoted by drought related to greater light use efficiency and dissipation of excitation energy in PSII antennae (Lima and Lobato, 2017; Hu et al., 2019). Foliar BRs sprays increased qP as a result of higher light absorption and flow of electrons accepted by plastoquinone, causing a high quantum efficiency of PSII and electron transport rate (Thussagunpanit et al., 2015; Lima and Lobato, 2017). The exogenous application of DI-31 promoted the reduction of NPQ in plants under drought stress. Pereira et al. (2019) report that reductions in NPQ values in plants treated with BRs indicate a decrease in the loss of photons mainly in the form of heat, by optimizing light use in photochemical processes.

The foliar application of DI-31 in lulo plants under drought stress generated increases in photosynthetic pigments (TChl and Cx + x). Treatments with DI-31 or BRs also caused higher photosynthetic pigments in *Arabidopsis thaliana* L. (Pérez-Borroto et al., 2021), soybean (Pereira et al., 2019), and sheepgrass (*Leymus chinensis* (Trin.) (Lv et al., 2020) plants exposed to water deficit. BRs analogues (DI-31) can inhibit the synthesis or activity of enzymes involved in the process of chlorophyll decomposition (Chl *b* reductase), promote a higher content of ascorbates, phenolic compounds, and tannic acid, and enhance the activity of antioxidant enzymes (Hussain et al., 2020). BRs also participate in the regulation of chlorophyll biosynthesis and decrease lipid peroxidation (Thussagunpanit et al., 2015; Sadura and Janeczko, 2018).

MDA production is the product of lipid peroxidation of membranes and has been considered a direct indicator of membrane damage under drought conditions (Yuan et al., 2010). Tani et al. (2018) recorded a significantly higher MDA content of eggplant (*Solanum melongena* L.) plants exposed to water stress. In this study, an increased MDA content was also found in lulo plants subjected to drought stress without the application of DI-31. Exogenous sprays of analogue BRs cause lower membrane lipid peroxidation (lower MDA) since this molecule favors the antioxidant system and improves the structure and stability of membranes (Yuan et al., 2010; Barros Junior et al., 2020; Hussain et al., 2020). Chá vez – Arias et al., 2020 also observed a decrease in the accumulation of MDA after the foliar application of DI-31 in cape gooseberry (*Physalis peruviana* L.) plants under water stress conditions.

BRs and analogues such as DI-31have been reported to improve plant responses to drought through the expression of genes involved in the tolerance mechanism to this abiotic stress (Serna et al., 2012a; Ahanger et al., 2018; Pérez-Borroto et al., 2021). Resilience to drought conditions in lulo plants after exogenous application of DI-31 may be related to the improvement of the activity of the major ROS-scavenging molecules (enzymatic and non-enzymatic) (Serna et al., 2012b; Ahanger et al., 2018; Pérez-Borroto et al., 2021). Additionally, BRs analogues may induce the expression of genes of proline synthesis that promote a higher proline (Yadav et al., 2012; Fu et al., 2019), and promote the elimination of ROS, the stability of plant cell membrane, and plant water status (Yadav et al., 2012; Vardhini and Anjum, 2015; Pérez-Borroto et al., 2021).

**Figure 8:**
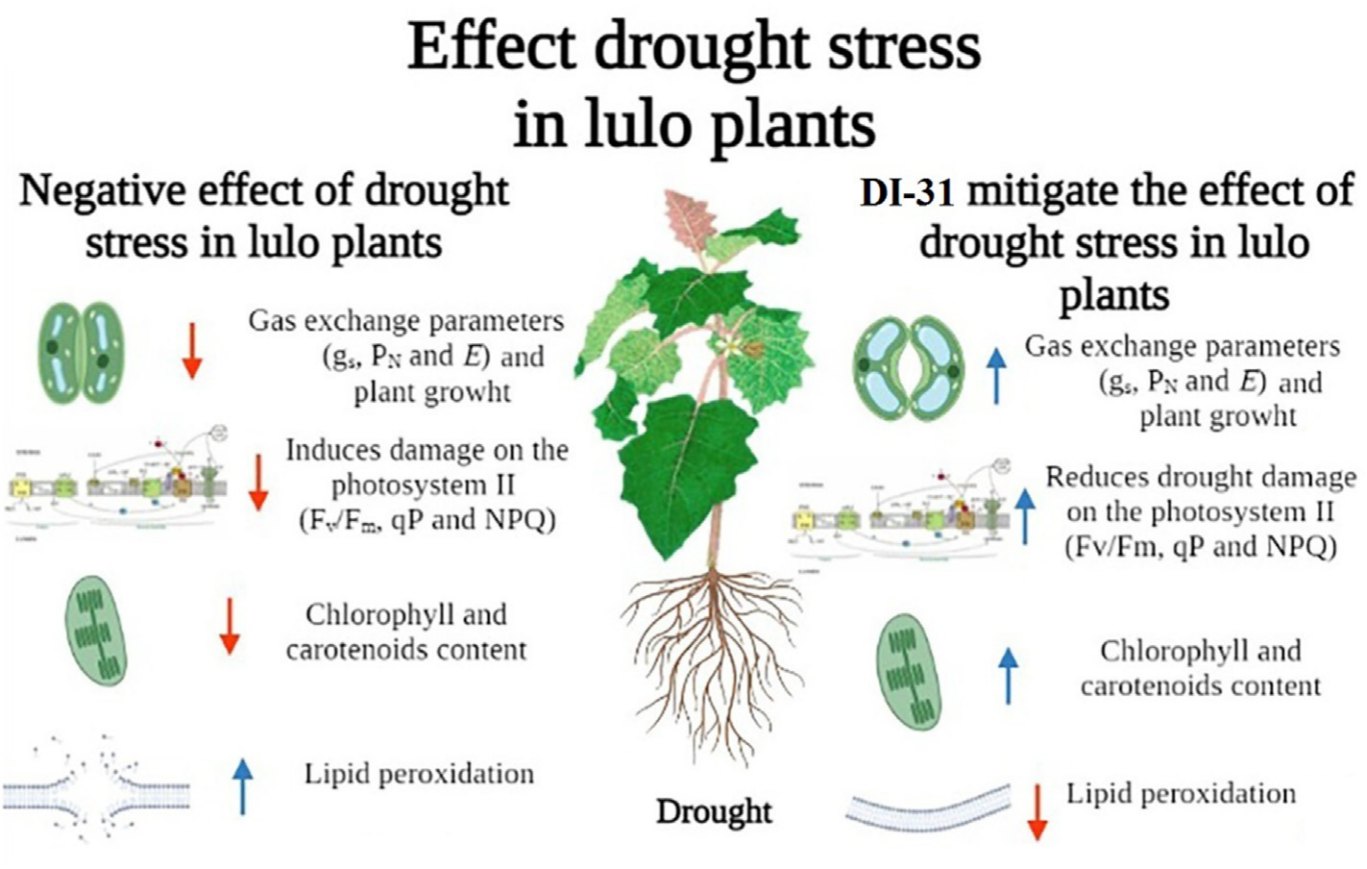
Concept model of the impact of the drought stress and foliar brassinosteroid analogue (DI-31) sprays in lulo plants. Red arrows and blue arrows indicate the negative or positive effect of interaction between drought stress and foliar DI-31 applications on the physiological and biochemical responses, respectively. g_s_: stomatal conductance; P_*N*_: net photosynthesis rate; *E*: transpiration; F_v_/F_m_: maximum quantum efficiency of PSII; qP: photochemical quenching; NPQ: non-photochemical quenching.

In Colombia, studies on lulo have been recently focused on the evaluation of plant responses to environmental stresses (waterlogging and light) using biochemical, morphoanatomical, or physiological parameters (Medina et al., 2006; Cardona et al., 2016; Sá nchez-Reinoso et al., 2019). However, the use of techniques to manage abiotic stress in lulo has been studied for waterlogging conditions in the country (Fló rez – Velasco et al., 2015). For this reason, foliar BRs analogue (DI-31) sprays constitute a suitable crop management technique since this molecule can stimulate the production of growth-promoting hormones (auxins and gibberellins). The application of this analogue can also induce differentially expressed genes related to drought tolerance, enhancing physiological and biochemical responses under drought conditions (Jiroutova et al., 2018; Huang et al., 2020; Díaz et al., 2021). Foliar DI-31 applications increased the tolerance (low DQE and high RTI) of lulo plants and enhanced the physiological and biochemical responses of plants subjected to drought stress conditions. The principal responses of lulo plants under drought were a decrease in leaf gas exchange parameters (P_N_, *g*_*s,*_ and transpiration), RWC, total chlorophyll, and carotenoid content. Additionally, plants showed increased PSII damage (lower values of chlorophyll fluorescence parameters such as F_v_/F_m_ ratio and qP, and higher NPQ values), and membrane lipid peroxidation. In contrast, these negative effects were ameliorated when lulo plants were foliar treated with BR (Figure 8).

## 5 Conclusion

In summary, this study revealed that this species shows a high susceptibility to drought mainly related to lower values of photosynthetic rate and water status. However, foliar BRs analogue (DI-31) applications, mainly at concentrations of 4 or 8 mL·L^−1^, helped plants cope with the adverse effects by improving physiological (leaf photosynthesis, photochemical efficiency of PSII, and plant growth) and biochemical [total chlorophyll and carotenoids concentration, and lipid peroxidation (malondialdehyde)] variables. Foliar DI-31 sprays can be a tool for managing water stress conditions because this plant hormone can generate a beneficial effect, helping lulo acclimation under low rainfall periods in producing areas. The results of this research allow us to go deeper into the agronomic management techniques to face water stress conditions generated by climate change and variability scenarios in Andean fruit crops.

## Declarations

### Author contribution statement

Cristian Camilo Castañeda-Murillo; Javier Gustavo Rojas-Ortiz; Alefsi David SánchezReinoso: Performed the experiments.

Cristhian Camilo Chávez-Arias: Conceived and designed the experiments; Analyzed and interpreted the data; Wrote the paper.

Hermann Restrepo-Díaz: Conceived and designed the experiments; Contributed reagents, materials, analysis tools or data; Wrote the paper.

### Funding statement

This research did not receive any specific grant from funding agencies in the public, commercial, or not-for-profit sectors.

### Data availability statement

The authors do not have permission to share data.

### Declaration of interests statement

The authors declare no conflict of interest.

### Additional information

No additional information is available for this paper.
